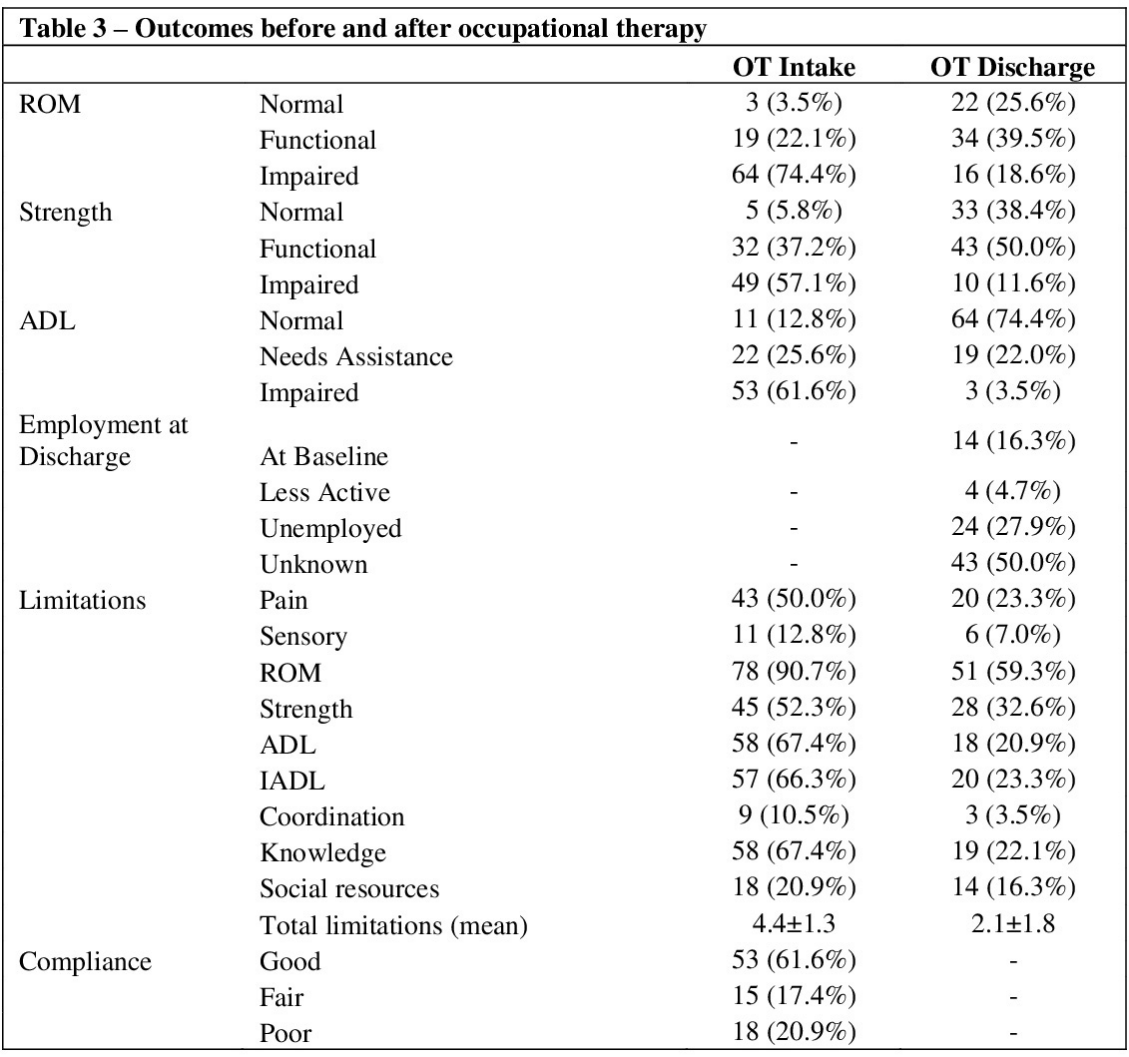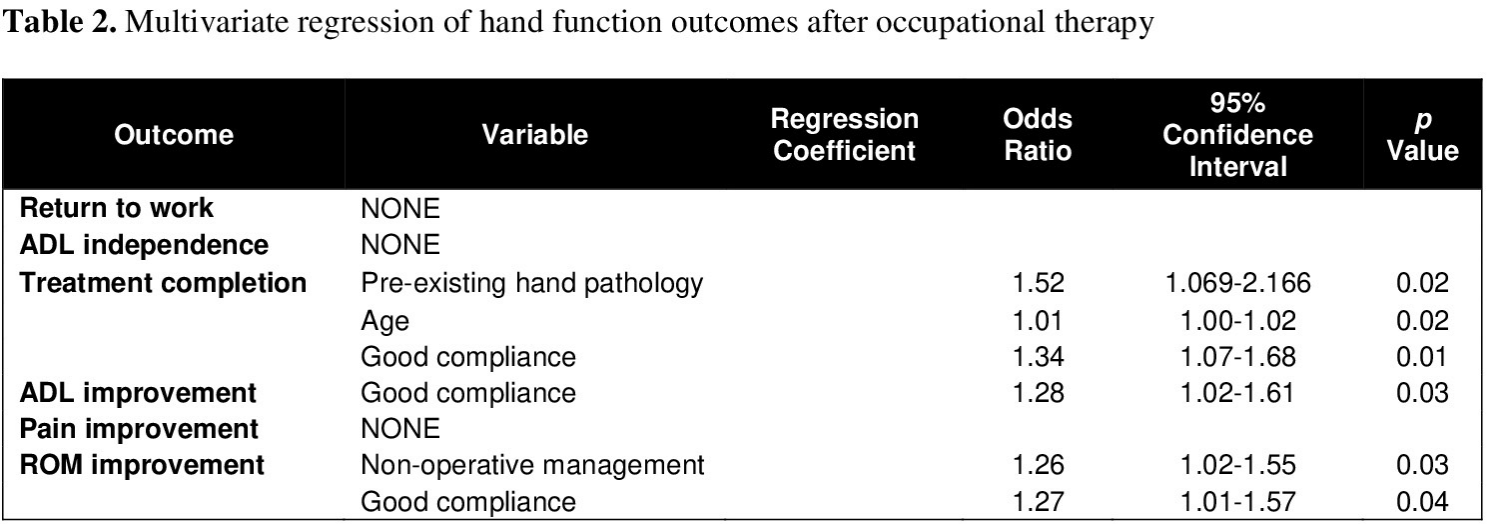# 24 Occupational Therapy Improves Range of Motion and Functional Independence in Patients with Hand Burns

**DOI:** 10.1093/jbcr/iraf019.024

**Published:** 2025-04-01

**Authors:** Artur Manasyan, Katherine Choi, Christopher Pham, Dawn Kurakazu, Zachary Collier, Justin Gillenwater

**Affiliations:** University of California Keck School of Medicine; Division of Plastic and Reconstructive Surgery, Geffen School of Medicine; Division of Plastic and Reconstructive Surgery, Keck School of Medicine; Los Angeles General Medical Center; Division of Plastic and Reconstructive Surgery, Keck School of Medicine; Los Angeles General Medical Center

## Abstract

**Introduction:**

Hands are the most commonly burned body part, and restoration of function is a paramount goal of treatment. Sparse data exists on functional outcomes of patients receiving outpatient therapy after admission with hand burns. There are few studies which have evaluated the effectiveness of long-term outpatient hand therapy on hand joint range of motion, activities of daily living (ADL) status, pain, and ability to return to work after burn injury.

**Methods:**

Adult patients with hand burns admitted to a single American Burn Association verified burn center from January 2015 to June 2024 with properly documented outpatient hand therapy follow-up were included. Patient demographics, injury variables, interventions, and long-term outcomes were evaluated. The effects of patient demographics and interventions on outcomes were evaluated with descriptive statistics and multivariable logistic regression.

**Results:**

86 patients were identified who consistently presented for outpatient OT. Median age was 41.0 (IQR 31-51) years, 63% (n=54) were male, and median TBSA was 10% (IQR 1.6-22.8). Surgical management with excision and sheet grafting was required in 74.4% (n=64), and mean time-to-surgery was 10.9±45.7 days from injury. Contractures occurred in 45.4% (n=39), and 29.1% (n=25) had hypertrophic scarring. Median OT follow up was 28.8±24.1) weeks. As of the last OT evaluation, 74.4% (n=64) had independent ADL function, 22% (n=19) required assistance, and 3.5% (n=3) were poorly functioning. Likewise, 25.6% (n=22) had normal ROM, 39.5% (n=34) were within functional limits, and 18.6% (n=16) had poor ROM. 75.6% (n=65) demonstrated improvement of ADL function, and 72.1% (n=62) had improvement of ROM. At OT intake, 50% (n=43) reported pain as a major limitation, but by the end of therapy, only 23% (n=20) were limited by pain. Those with pre-existing hand pathology and older age were more likely to complete therapy to completion (OR=1.52 and 1.01, CI 1.1-2.2 and 1.1-1.7, p=0.02 for both), and those who did not require surgery and had good compliance as rated by therapists had significantly increased likelihood of ROM improvement (OR=1.26 and 1.27, CI 1.0-1.6 and 1.0-1.6, p=0.03 and 0.04).

**Conclusions:**

Most patients referred for hand therapy after burn injury return to functional independence and have functional ROM. Further research should take a prospective approach to evaluate the efficacy of hand occupational therapy after burns, focusing on a wider range of psychosocial outcomes.

**Applicability of Research to Practice:**

Non-operative management and longer duration of OT could result in better long-term outcomes. Burn surgeons should actively involve occupational therapists for hand burns, as this collaboration significantly improves patient outcomes.

**Funding for the Study:**

N/A